# Spatial and temporal analysis of tuberculosis in an area of social inequality in Northeast Brazil

**DOI:** 10.1186/s12889-019-7224-0

**Published:** 2019-07-04

**Authors:** Shirley Verônica Melo Almeida Lima, Allan Dantas dos Santos, Andrezza Marques Duque, Marco Aurélio de Oliveira Goes, Marcus Valerius da Silva Peixoto, Damião da Conceição Araújo, Caíque Jordan Nunes Ribeiro, Márcio Bezerra Santos, Karina Conceição Gomes Machado de Araújo, Marco Antônio Prado Nunes

**Affiliations:** 1 Program in Health Sciences, Federal University of Sergipe, Brazil Cláudio Batista, s/n, Cidade Nova, Aracaju, SE 49060-108 Brazil; 2 Nursing Department, Federal University of Sergipe, Avenida Universitária Marcelo Deda Chagas, 330, Lagarto, SE 49.400-000 Brazil; 3 Department of Health education, Federal University of Sergipe, Avenida Universitária Marcelo Deda Chagas 330, Lagarto, SE 49.400-000 Brazil

**Keywords:** Spatial analysis, Prevention and control, Tuberculosis, Epidemiology, Time series studies

## Abstract

**Background:**

Tuberculosis is an infectious disease caused by *Mycobacterium tuberculosis*. It is a disease known worldwide for its vulnerability factors, magnitude and mortality. The objective of the study was to analyze the spatial and temporal dynamics of TB in the area of social inequality in northeast Brazil between the years 2001 and 2016.

**Methods:**

An ecological time series study with the use of spatial analysis techniques was carried out from 2001 to 2016. The units of analysis were the 75 municipalities in the state of Sergipe. Data from the Notification of Injury Information System were used. For the construction of the maps, the cartographic base of the state of Sergipe, obtained at the Instituto Brasileiro de Geografia e Estatística, was used. Georeferenced data were analysed using TerraView 4.2.2 software (Instituto Nacional de Pesquisas Espaciais) and QGis 2.18.2 (Open Source Geospatial Foundation). Spatial analyses included the empirical Bayesian model and the global and local Moran indices. The time trend analyses were performed by the software Joinpoint Regression, Version 4.5.0.1, with the variables of sex, age, cure and abandonment**.**

**Results:**

There was an increasing trend of tuberculosis cases in patients under 20 years old and 20–39 years old, especially in males. Cured cases showed a decreasing trend, and cases of treatment withdrawal were stationary. A spatial dependence was observed in almost all analysed territories but with different concentrations. Significant spatial correlations with the formation of clusters in the southeast and northeast of the state were observed. The probability of illness among municipalities was determined not to occur in a random way.

**Conclusion:**

The identification of risk areas and priority groups can help health planning by refining the focus of attention to tuberculosis control. Understanding the epidemiological, spatial and temporal dynamics of tuberculosis can allow for improved targeting of strategies for disease prevention and control.

## Highlights

- We studied standardized municipality tuberculosis case notification rates in an area of social inequality in Brazil.
- Between 2001 and 2016, the study identified increasing trends of TB in Sergipe with inequalities between age groups and sex.
- The cure rate presented a worrying decline, countering the steady trend of treatment abandonment.
- Tuberculosis rates were strongly positively spatially autocorrelated.
- Higher rates were associated with population density and socioeconomic conditions.

## Background

Tuberculosis (TB) is an infectious disease caused by an intracellular pathogen called *Mycobacterium tuberculosis*. It is a disease known worldwide for its vulnerability, magnitude and morbidity factors. TB is still a serious global public health problem, and it is in the top 10 causes of mortality in underdeveloped countries [1].

Brazil has been undergoing a process of epidemiological transition, with the increase of chronic and non-transmissible diseases and the reduction of infectious and parasitic diseases [2]. However, TB remains on the horizon of public health, mainly due to its persistence and high rates in communities living in poverty and on the margins of social exclusion [3].

In the world, it is estimated that 10.4 million people became ill with TB in 2015. The highest incidence rates were reported on the Asian and African continents [1]. However, Brazil is also considered an endemic country for TB. In 2018, the country presented an incidence rate of 33.5 cases per 100,000 inhabitants and Sergipe, with an incidence rate of 28.8/100,000, had the fourth highest incidence rate of TB in the northeast region, despite the global reduction target of 10/100,000 inhabitants as a strategy for eliminating the disease [4, 5].

The probability of an infected person developing the disease depends on several factors, including social determinants [6], socioeconomic characteristics and coinfection by the human immunodeficiency virus (HIV), which are risk factors in the epidemiological chain of TB transmission, infection and dissemination [7]. All these factors contribute significantly to the high morbidity and mortality rates of patients [8].

It is critical that information about the operational and clinical characteristics of TB are disseminated in order to understand the extent and severity of the disease and its impact on public health. Spatial and temporal approaches help to better understand the distribution, dynamics and risk of diseases, especially TB and its variations over time.

The use of database helps studies that intend to analyse the manifestation of the disease over time. Studies of this nature can predict the probability of events based on continuous data streams [9]. In addition, the use of geoprocessing techniques with geographic information systems (GIS) combined with spatial statistics allows for the incorporation of distinct variables, especially demographic and social variables, in health studies [10, 11], making it possible to generate hypotheses regarding the transmission of diseases in different populations [12, 13].

Sergipe, an area located in northeast Brazil, presents a high incidence rate [5], a fact that worries us as researchers of the subject. The maintenance of high rates and having practically all the territory affected by the disease motivated us to study this region. The impacts on the health of the population and the frailties faced in the prevention and control of TB also justify the relevance of investigations. In this sense, the objective of the study was to analyse the spatial and temporal dynamics of TB in the area of social inequality in northeast Brazil between the years 2001 and 2016.

## Method

### Design and location of study

This was an ecological study with temporal and spatial components. We used spatial analysis techniques and time series from 2001 to 2016 in the state of Sergipe, considering the 75 municipalities as units of analysis.

The state of Sergipe has an estimated population of 2,265,779 inhabitants and a population density of 94.3 inhabitants per km^2^ [14]. The state is divided into 75 municipalities with areas that comprise a population with diverse socioeconomic and environmental characteristics [15].

### Sample, data collection and outcome

The sample consisted of all confirmed cases of TB, considering the International Classification of Diseases 10th revision (ICD-10). Data recorded in the Notification of Injury Information System (SINAN), made available by the State Health Department of Sergipe, were used. The outcome was the incidence of TB in Sergipe.

### Epidemiological data analysis

The epidemiological and operational indicators analysed in this study were the annual incidence rates, sex, age group (classified as less than 19, 20–39, 40–59 and 60 years or more). The frequency of abandonment and cure was determined for analysis of time trends. Data were stored in Microsoft Excel spreadsheets (Windows version 2013, Microsoft Corporation; Redmond, WA, EUA).

We used the Joinpoint Regression Program (Statistical Methodology and Applications Branch, Surveillance Research Program of the National Cancer Institute, Bethesda, MD, EUA, June 2017, Version 4.5.0.1) to calculate the temporal trends of TB frequency, abandonment and cure of cases recorded with a model based on the assumption of a minimum number of points (Joinpoint). A logarithmic linear regression model was also performed with the Monte Carlo permutation test [15–17]. The annual percent change (APC) was calculated and a 95% confidence interval was adopted. 95%).

### Spatial analysis

For construction of the spatial analysis maps, we used the cartographic base of the state of Sergipe, available in the electronic database of the Instituto Brasileiro de Geografia e Estatística (IBGE) [14]. The cartographic projection corresponds to the SIRGAS 2000 Universal Reference System, and the georeferenced data were analysed by the TerraView software (Instituto Nacional de Pesquisas Espaciais, INPE, SP, BR, Version 4.2.2) and QGIS (Open Source Geospatial Foundation, OSGeo, CHI, US, Version 2.18.2). We also used the Bayesian estimator to minimize the instability caused by the random fluctuation of cases and to smooth the standardized rates by applying weighted averages and creating a third corrected rate [17].

The spatial analysis maps, constructed from the annual incidence coefficients, illustrated the mean number of TB cases recorded in each of the five years by municipality, using a moving average between periods which was then divided by the estimated population of the year of the medium and multiplied by 100,000. This process resulted in six maps with new estimated taxa, which were analysed by spatial autocorrelation approaches.

The empirical Bayesian local rate illustrated a correction of the multiplicative rate equal to 100,000, taking into account the population at risk and the number of cases for each five years analysed. To estimate the spatial variability in the data analysis, a proximity matrix was constructed, in which adjacent municipalities were awarded the value 1 (one), and those that did not present adjacent border geometries were awarded the value 0 (zero) [18].

The Global Moran’s Index was used to evaluate the spatial autocorrelation of the TB incidence rate in the geographic space analysed, varying between − 1 and + 1. Values close to 0 indicate spatial randomness; values between 0 and + 1 indicate positive spatial autocorrelation; and values between − 1 and 0 indicate negative spatial autocorrelation. The level of significance of 5% was used [18]. The analysis by the Global Moran’s Index investigates whether the distribution occurs randomly or follows some pattern of occurrence in space [19].

The spatial dependence showed by the Local Moran’s Index provides the local indicator of spatial association (LISA) for the detection of regions with significant spatial correlation and clustering measures [20].

The Moran map was used to indicate the critical and transitional areas that were statistically significant, signalling categories of the clusters and their relations with the neighbours. This analysis verifies the existence of spatial dependence and risk patterns for sickness with the creation of spatial quadrants, Q1 (high/high) and Q2 (low/low), which indicate municipalities with similar values as those of their neighbours, and Q3 (high/low) and Q4 (low/high) with different values. The first two categories represent areas of agreement with aggregates of positive spatial association, and the last two categories represent areas of transition with aggregates of negative spatial association [21, 22].

### Ethical aspects

The research was approved by the Committee of Ethics and Research with Humans, under protocol n° 1,768,049. The rules of the Helsinki Convention were followed.

## Results

A total of 10,883 TB cases were registered in the state of Sergipe from 2001 to 2016. Of those, 9166 were new cases, 559 were relapses, 738 were readmissions after abandonment, 412 were transfers from other states and eight were recorded after death.

**Fig. 1:**
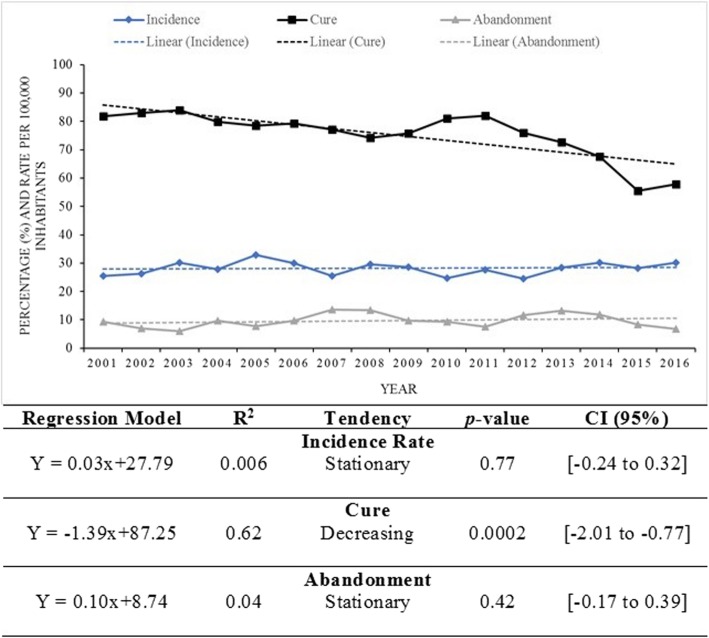
Temporal trend of TB cases by incidence rate, cure and treatment abandonment, Sergipe, Brazil (2001–2016)

The mean TB incidence rate in the study period was 28.35 per 100.000 inhabitants (minimum of 24.49 and maximum of 32.98). The incidence rate also presented a stationary trend during that period (Fig. 1). Similarly, the treatment abandonment rate showed a stationary trend. However, the percentage of cured patients presented a decreasing trend in this period (81.78% in 2001 to 57.81% in 2016).

The specific temporal trend of TB cases by age group and sex was significantly increased in males and in the 20–39 age group (Table 1). In both sexes, the periods between 2001 and 2005 and between 2010 and 2016 showed statistically significant elevation.

**Table 1:** Temporal trend of TB by sex and age group in the state of Sergipe, Brazil (2001–2016)

	Segment	Period	APC	CI 95%	
				Minimum	Maximum
Male					
< 19	1	2001–2016	0.6	−2.9	4.3
20–39	1	2001–2003	21.8	− 9.2	63.2
	2	2003–2010	− 3.4	−7.4	0.8
	3	2010–2016	7.7*	3.6	11.9
40–59	1	2001–2016	−2.3*	−3.6	− 1.0
60 e +	1	2001–2016	−1.7	−4.0	0.6
Female					
< 19	1	2001–2016	0.6	−2.0	3.4
20–39	1	2001–2016	− 2.4*	− 4.1	− 0.7
40–59	1	2001–2016	− 2.4*	−4.4	− 0.3
60 e +	1	2001–2016	− 0.2	−2.5	2.1
Total					
< 19	1	2001–2016	0.8	− 1.7	3.3
20–39	1	2001–2005	6.7*	0.1	13.7
	2	2005–2010	− 6.5*	−12.0	− 0.7
	3	2010–2016	6.3*	3.1	9.5
40–59	1	2001–2016	−2.4*	− 3.6	− 1.2
60 e +	1	2001–2016	− 1.2	− 3.0	0.6

*APC* annual percent change, *CI* confidence interval, * = significant for *p-*value < than 0.05

**Fig. 2:**
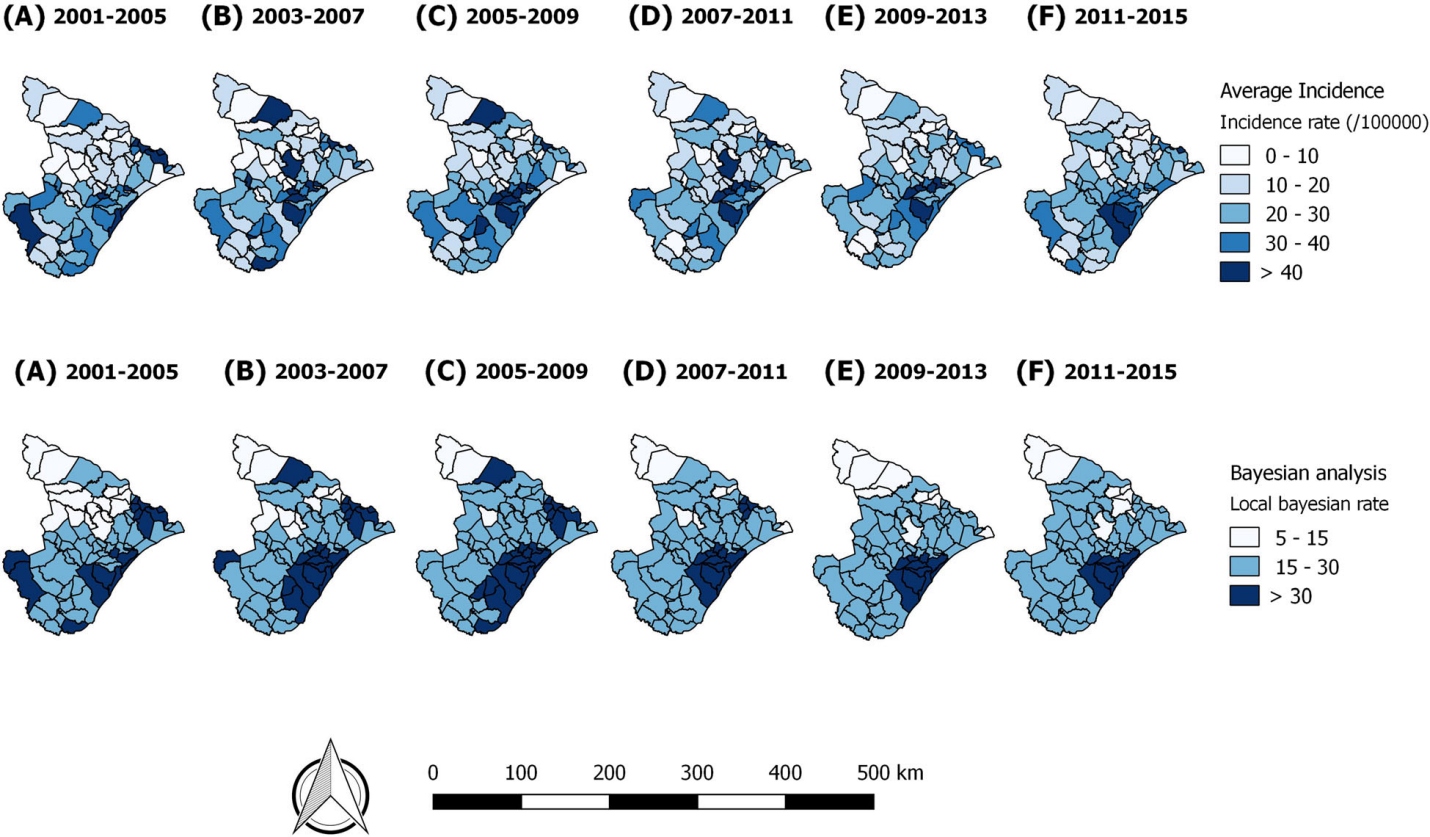
Mean incidence rates of TB and rates corrected by empirical Bayesian model, Sergipe, Brazil (2001–2015)

For the analysis of crude incidence rate, a high concentration of diffusely distributed disease was observed in several regions of the state, with intensity in the southeast region (Fig. 2). For the Bayesian maps, clusters of risk were visualized in the Northeast of the state (between 2001 to 2009) and the Southeast (between 2001 and 2015) Table 2.

**Table 2:** Global Moran’s Index of the mean incidence rates for TB, Sergipe, Brazil (2001–2015)

Period	Incidence Rate		
	I	*p*-value	min.-max.
2001–2005	0.283	0.005	0.0–70.9
2003–2007	0.056	0.205	0.0–65.2
2005–2009	0.137	0.046	0.0–83.6
2007–2011	0.108	0.076	0.0–55.3
2009–2013	0.318	0.001	0.0–53.0
2011–2015	0.256	0.003	0.0–60.5

I - Global Moran’s Index; min-max: minimum and maximum

**Fig. 3:**
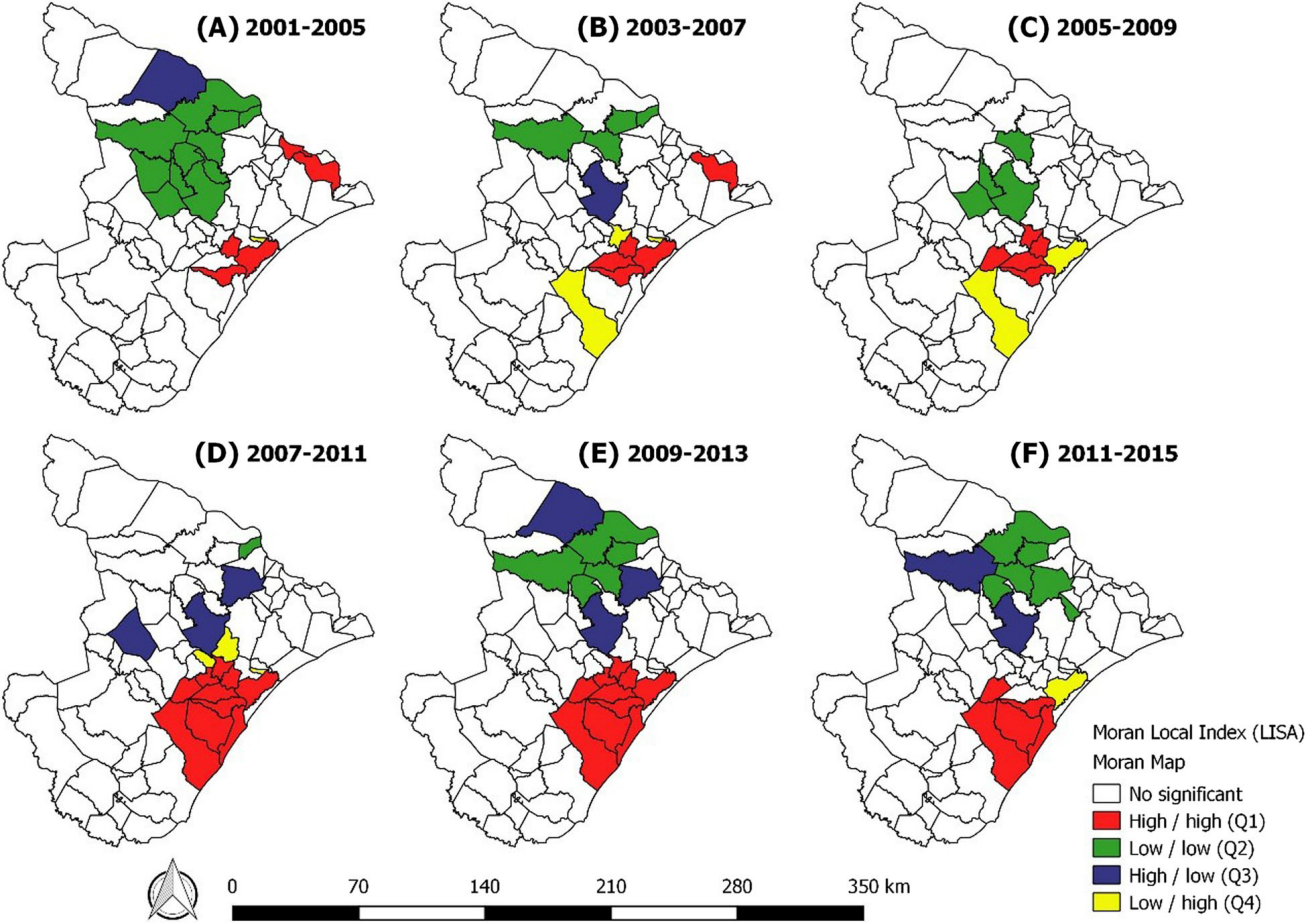
Moran Local Index with Moran map analysis using the average incidence for TB, Sergipe, Brazil (2001–2015)

The Moran map analysis allowed for the identification of the municipalities classified according to the level of significance of their local indexes. Two regions with agglomerates were identified: one in the southeast with high incidence rates (Q1) and the other in the north with the lowest incidence of the disease (Q2) (Fig. 3). The eastern region (2001–2005) presented three municipalities with high incidence rates and, over time, expanded to previously untouched regions in the central and southeast sections of the country.

The municipalities represented by Q4 indicate areas of transition of the disease, but they deserve attention because they are close to municipalities with a high incidence of TB (Fig. 3). Agglomerates classified with Q3 present a high incidence of TB and can interfere in neighbouring municipalities with a low incidence of TB.

**Fig. 4:**
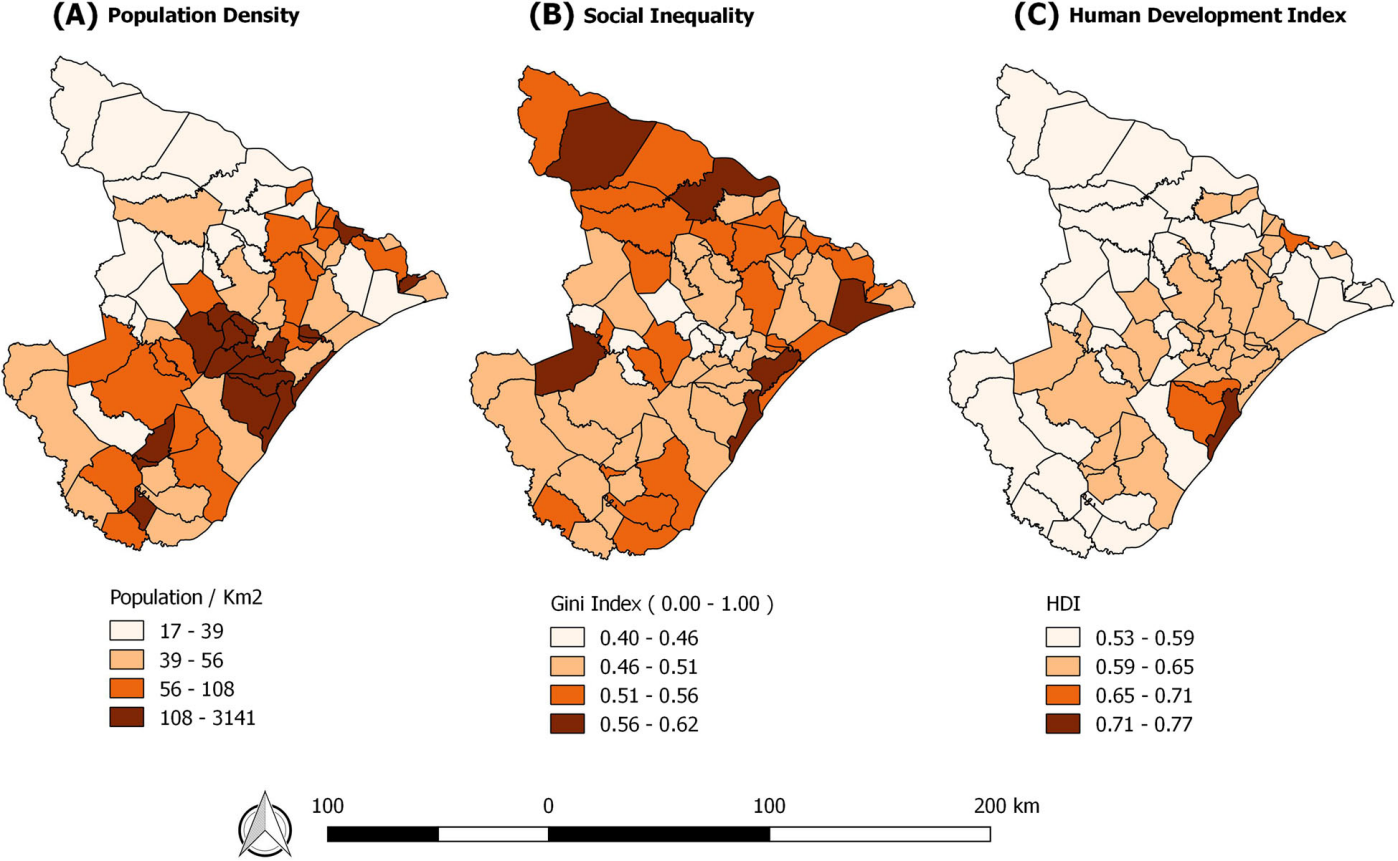
Socioeconomic indicators and population density, Sergipe, Brazil (2016)

Maps of social inequality and population density, as displayed with higher hue intensity, also indicate population clusters in Sergipe. The human development index showed that the southeast region is the most developed (Fig. 4).

## Discussion

TB still represents one of the most important infectious diseases in the world/ Even with all the advances made in its eradication, it is a disease that still presents alarming data on morbidity and mortality. In Brazil, TB has an endemic character [23], and over time, its occurrence has varied, presenting different risks in the regions of the country [24]. However, in spite of all the efforts and campaigns of the Ministry of Health to reduce the transmission and the occurrence of TB cases, the state of Sergipe did not show a reduction in the incidence rate between the years of 2001 and 2015. In addition, there are impressive data on the decrease in the cure rate of these patients.

The decreasing trend in patient cure rates is undoubtedly the most alarming data in our study and reveals a bleak and concerning epidemiological scenario for TB in the state of Sergipe. This trend may be justified with withdrawal episodes, which makes the patients resistant to treatment.

In addition, there was also a growing trend for TB cases among men, mainly in the age group between 20 and 39 years of age. This trend was also observed in other studies [25–27] that corroborate our findings. These data signal an alert situation, since they may indicate a high transmission in the population, since most of this group is possibly ill due to recent infections. This finding may also be associated with delays in diagnosis, social factors that make diagnosis and control difficult, and areas with insufficient screening measures [28].

The use of the Geographic Information Systems to identify areas of risk and active transmission of TB has been noted as an effective tool for the monitoring and elaboration of health strategies, especially in regions with high incidence rates and aspects of social vulnerability [29, 30].

The spatial dynamics of the disease has shown a distinct behavior, according to the regions of the state of Sergipe. This finding reflects different risks for illness, showing a high incidence mainly in the South and Southeast regions and show changes in the pattern of the disease over the years. In 2001–2005, TB was concentrated in regions with low economic growth, while in 2011–2015 there was a higher prevalence in more urbanized areas. One hypothesis for this was the migratory process of people from the north to the southeast region of the state looking for better living conditions in the more economically developed areas.

Currently, the peripheries of large urban centres (especially the favelas) present clear economic and social discrepancies. Accelerated urbanization and inadequate infrastructure, common to developing countries, explain an important part of the current epidemiological profile [2]. It is known that TB is closely associated with poverty, poor living conditions and housing and human agglomeration [31, 32].

The disorderly growth of metropolitan cities and the formation of pockets of poverty may leave regions even more susceptible to acquiring the infection. The increase in TB transmission is evident, because the existence of people without the correct treatment contributes to the spread of the bacillus in the population [33, 34].

To Brazil has own characteristics according to the political, social and scientific context. Over the years, important improvements were seen in the therapeutic regimen, the diagnosis process, the treatment time, the identification of new cases and the evaluation of the contacts, and the follow up and support of the health services. However, new historical events emerged and continued to maintain TB as a worldwide epidemic and with difficult control, such as social inequality; the advent of AIDS, the multi-resistance, the aging population and migratory movements [35–37].

In this scenario, overly programmatic health services limited the priority themes and did not allow a truly decentralized dynamic of municipal health services, hampering the intelligent confrontation of issues [38]. These individual and ecological effects have had an important role in the change TB spatial patterns in Brazil and especially in Sergipe.

The correction of the TB incidence rate by the local Bayesian method allowed for the minimization of possible distortions of the variability provided by the calculation of gross rates due to the size of the population at risk. The rate adjustment is more consistent from the epidemiological point of view, since it showed a more uniform pattern of risk, although it presents high and worrying values, since they extrapolate the global goal and the current national situation.

The Moran map pointed to the Southeast region with a higher concentration of cases and significant spatial dependence. Municipal agglomerations in the Southeast region presented similar behaviours for TB, which may induce, in the near future, the continuous spread of the disease and new infections, even in neighbouring municipalities. Areas with a high prevalence of TB over time suggest the existence of a substantial number of latent infections in the population [39]. Identifying the main risk areas based on geographic units is necessary for the formulation of equitable public policies [40].

The identification of clusters indicated an expanded epidemiological view of the areas at greatest risk of transmission. Spatial analyses indicated future, possibly avoidable, risks in areas that require further development in health actions and consolidation of surveillance measures. Health systems need to respond better and faster to the challenges of a changing world [41]. In this perspective, health promotion and prevention actions should be planned according to the real needs of the exposed population.

TB is a complex disease that presents control difficulties and is easily spread throughout the world. It is possible to affirm that there is a relation between territorial space and transmission of TB; thus, to understand these dynamics, it is important to move beyond individual level analysis to spatial dimensions in order to receive important input for targeted intervention [42].

When considering the transcendence of the disease and its geographical exposure, we can suggest alert situations, as they imply greater exposure and possible illness. When observing the main areas of risk, one can observe relations with the population density, since areas with high infection intensity coincided with areas of high population concentration. It is therefore reiterated that prevention and control strategies can be established through spatially dependent relationships between TB and its risk factors [43].

The social context certainly undermines the space relations established in this study. It is clear that regions with higher levels of human development also show large income inequalities as seen in Fig. 4. The authors believe that although there is an economic development, large pockets of poverty still prevail in cities with accelerated urbanization. The research provides evidence of how the social determination of municipalities may be related to the increased incidence of TB. This awareness must be present among policy makers so that the proposed improvements are both intersectoral and health service improvements.

Territory-based surveillance replicates more precise disease monitoring [44], and TB incidence studies will continue to provide the best method to directly measure the disease burden and identify actions necessary for its reduction [1].

The authors understand that there are limitations in the method, such as not differentiating patients deprived of their freedom and not including patients with latent tuberculosis. There are limitations also when analysed only ecological characteristics and not individual characteristics and their risk factors. However, it is believed that the results can bring significant benefits to the control of the disease in Brazil, creating new health practices and strengthening social public policies for the population.

## Conclusion

The study identified increasing trends of TB in Sergipe with difference between age groups and sex. The cure rate presented a worrying decline, countering the stable trend of treatment abandonment. The spatial findings showed the existence of TB throughout the territory and emphasized space as influential in the onset of the disease. Positive spatial autocorrelation was observed in the Southeast region, characterized by more urbanized municipalities with high population density.

The research expanded knowledge about social conditions and TB infection and strengthened evidence regarding the temporal and spatial dynamics of TB to improve prevention actions and control of the disease. The analysis elucidated discussions of social and structural health system problems, operations, and the need for possible strategic changes to address TB. Further research is needed to address populations younger than 20 years and over 60 years to better determine the environmental and social factors that interfere with the transmissibility of the disease in these groups.

## Data Availability

The datasets analyzed during the current study consisted of all confirmed cases of TB, considering the International Classification of Diseases 10th revision (ICD-10). Data recorded in the Notification of Injury Information System (SINAN), that supporting the results of this study are available from [State Health Department of Sergipe]. The data are, however, available by the authors upon reasonable request and with permission of [State Health Department of Sergipe]. For the construction of the spatial analysis maps, we used the cartographic base of the state of Sergipe, available in the electronic database of the Instituto Brasileiro de Geografia e Estatística (IBGE).

## Abbreviations

APC: Annual percent change
CI: Confidence interval
GIS: Geographic information systems
HIV: Human immunodeficiency virus
I: Global moran’s index
IBGE: Instituto brasileiro de geografia e estatística
ICD-10: International classification of diseases 10th
LISA: Local indicator of spatial association
Q1: Quadrant 1
Q2: Quadrant 2
Q3: Quadrant 3
Q4: Quadrant 4
SINAN: Notification of injury information system
SIRGAS 2000: universal reference system
TB: Tuberculosis
